# Carnivore distribution across habitats in a central-European landscape: a camera trap study

**DOI:** 10.3897/zookeys.770.22554

**Published:** 2018-07-04

**Authors:** Klára Pyšková, Ondřej Kauzál, David Storch, Ivan Horáček, Jan Pergl, Petr Pyšek

**Affiliations:** 1 Department of Ecology, Faculty of Science, Charles University, Viničná 7, CZ-12844 Prague 2, Czech Republic; 2 Institute of Botany, Department of Invasion Ecology, The Czech Academy of Sciences, CZ-25243 Průhonice, Czech Republic; 3 Center for Theoretical Study, Charles University and The Czech Academy of Sciences, Jilská 1, CZ-11000, Prague 1, Czech Republic; 4 Department of Zoology, Faculty of Science, Charles University, Viničná 7, CZ-12844 Prague 2, Czech Republic

**Keywords:** camera trap, Elbe River catchment, central Bohemia, circadian activity, ecology, seasonal dynamics

## Abstract

Quantitative data on local variation in patterns of occurrence of common carnivore species, such as the red fox, European badger, or martens in central Europe are largely missing. We conducted a study focusing on carnivore ecology and distribution in a cultural landscape with the use of modern technology. We placed 73 automated infra-red camera traps into four different habitats differing in water availability and canopy cover (mixed forest, wetland, shrubby grassland and floodplain forest) in the Polabí region near Prague, Czech Republic. Each habitat was represented by three or four spatially isolated sites within which the camera traps were distributed. During the year of the study, we recorded nine carnivore species, including the non-native golden jackal. Habitats with the highest numbers of records pooled across all species were wetland (1279) and shrubby grassland (1014); fewer records were made in mixed (876) and floodplain forest (734). Habitat had a significant effect on the number of records of badger and marten, and a marginally significant effect on fox. In terms of seasonal dynamics, there were significant differences in the distribution of records among seasons in fox, marginally significant in least weasel, and the occurrence among seasons did not differ for badger and marten. In the summer, fox and marten were more active than expected by chance during the day, while the pattern was opposite in winter when they were more active during the night. Our findings on habitat preferences and circadian and seasonal activity provided the first quantitative data on patterns whose existence was assumed on the basis of conventional wisdom. Our study demonstrates the potential of a long-term monitoring approach based on infra-red camera traps. Generally, the rather frequent occurrence of recorded species indicates that most carnivore species are thriving in current central-European landscapes characterized by human-driven disturbances and urbanization.

## Introduction

In the last decade, field research of mammals has principally changed with the invention of automated camera traps, which are now becoming a standard monitoring tool (O’Connell et al. 2011, Burton et al. 2015). With the rapid technological advances, camera traps have been gaining more attention and popularity as they allow for the non-intrusive observation of animals and rapid and efficient collection of large data sets that are both unique and high quality. Mammals, and particularly carnivores, are a group of animals that are not easy to monitor due to their mobility and mostly nocturnal and crepuscular activity, and their intelligence and shyness. Camera traps allow insight into this hidden world without disturbing the observed organisms.

While most studies using camera traps have focused on a particular species, habitat type, activity or behaviour (e.g. Ahumada et al. 2011, Manzo et al. 2011, Braczkowski et al. 2016), complex studies addressing diversity, species composition and behaviour across habitat types are missing. In the Czech Republic, most attention has been paid to large and rare carnivore species, such as the Eurasian lynx (*Lynx lynx*) and the gray wolf (*Canis lupus*), iconic representatives of a charismatic group of animals and the focus of nature conservation (Baruš et al. 1989, Kutal et al. 2016, Kutal 2017). These carnivores began returning to this country in the 1990s from neighbouring regions and have established viable populations. In addition, camera traps proved useful for discovering and monitoring the presence and behaviour of species spreading from other regions, such as the golden jackal, *Canis aureus* (Pyšková et al. 2016). However, the more common carnivore species (such as martens, weasels, foxes and badgers) are largely neglected and to date have never become a target of systematic quantitative investigation using camera traps over a long period of time. Within the temperate zone such data are lacking completely not only for the Czech Republic, but for Europe as a whole. Moreover, the majority of literature sources on carnivore ecology and distribution in the Czech Republic are rather outdated (e.g. Mazák 1964, Baruš and Zejda 1981), based on information that is often anecdotal, and much of the quantitative data on these species’ distributions come from hunting statistics or questionnaires, which can suffer from various biases. Data on habitat preferences, seasonal and circadian activity, and presence of common carnivore species in the changing landscapes of central Europe are lacking. We know little about how these animals adapt to the heavily inhabited modern environment.

To contribute towards closing this gap and to provide the first basic quantitative insights into the patterns of carnivore distribution in typical central-European habitats, we (i) recorded the species richness and composition of carnivores in a typical temperate mosaic landscape, (ii) quantitatively compared carnivore presence in different habitats along the moisture and canopy-cover gradients, (iii) analysed the seasonal and circadian activity of the species in the course of a whole year, and (iv) identified any non-native species in the area studied.

## Methods

### Study area

**Figure 1.**
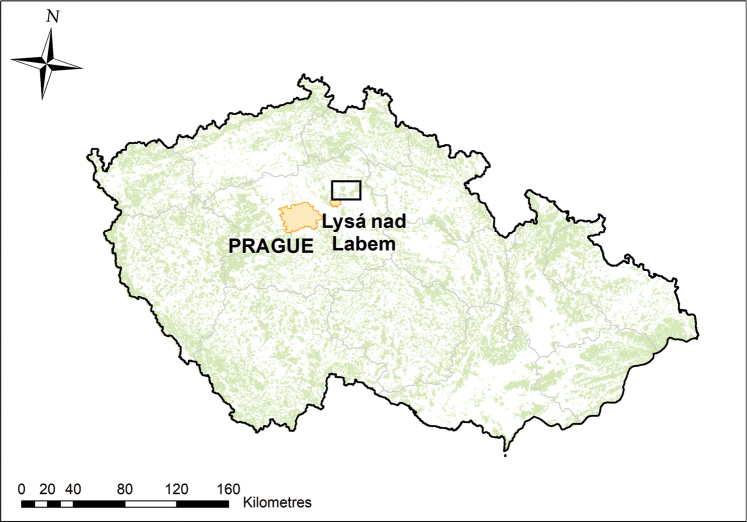
Location of the study area in central Bohemia, western part of the Czech Republic (black rectangle).

The study area was located ~30–40 km east of Prague in the Elbe River catchment, in the districts of Nymburk and Mladá Boleslav (Fig. 1). The area is quite heavily inhabited (114 and 123 inhabitants per km^2^, respectively; Český statistický úřad 2016) and agriculturally dense (70.2% of the central Bohemia district is covered by agricultural land, 23.9% by forest, 4.6% by artificial surfaces and 0.7% by water bodies; Corine Land Cover 2012, version 18.5.1 – CENIA 2012). We chose this region because it contains various types of habitats and represents a typical central-European landscape, consisting of a mosaic of human-made and seminatural habitats. The majority of the study area is at an altitude of < 200 m a.s.l., with a warm mild climate, annual average temperature of 8.5–9.0 °C and annual precipitation of 550 mm. The size of the study area, expressed as the landscape sections over which the 13 sites were distributed was ~200 km^2^. From the botanical perspective, the area belongs to thermophyticum, district of thermophilous flora with vegetation cover formed by oak and hornbeam forest, dry grassland and xerophilous shrub (Kaplan 2012). Recently, part of the area was used for reintroduction of ungulates that were driven to extinction by past human activities, such as a breed of domestic cattle resembling the auroch (*Bos primigenius*), the Exmoor pony as a breed resembling the wild horse (*Equus ferus*), and European bison (*Bison bonasus*; Stokstad 2015).

### Habitats

**Figure 2.**
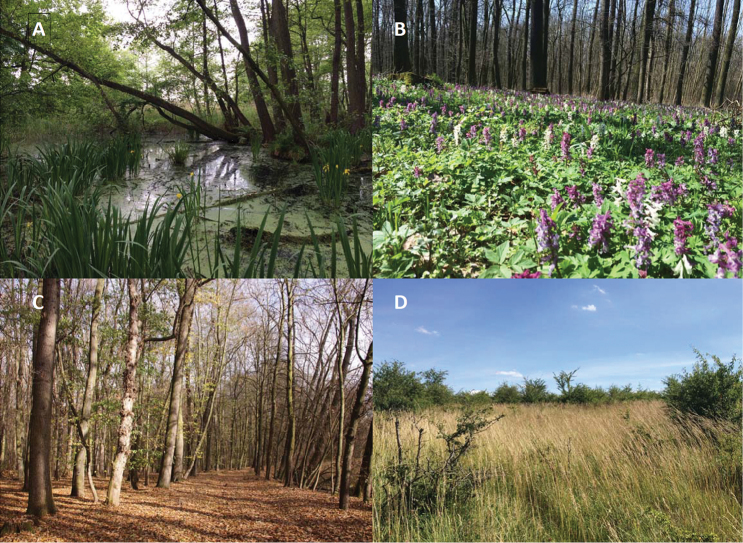
Habitat types studied. **A** wetland **B** floodplain forest **C** mixed forest **D** shrubby grassland (see text for description). Photo credits: Klára Pyšková

**Figure 3.**
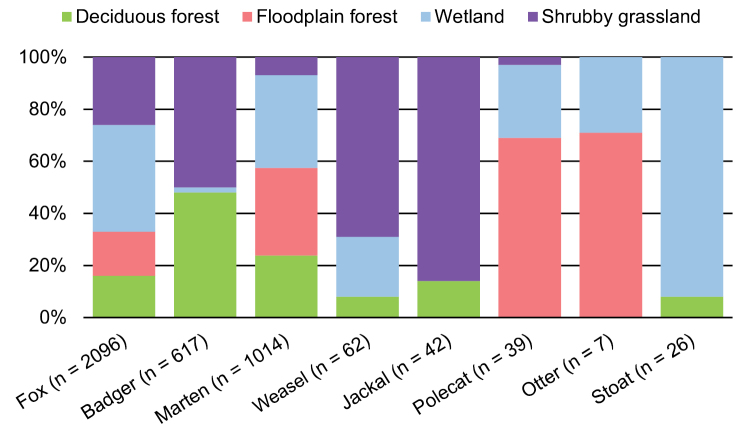
Habitat preferences of the carnivores studied; the figures are percentages of the total number of standardized daily records as recorded in each habitat.

The habitats chosen for this project were wetland, floodplain forest, mixed forest and a shrubby grassland (steppe), forming a distinct moisture- and canopy-openness gradient (see Fig. 2):

(i) Wetland habitat, the wetter alternative of the open biotope, had a high groundwater level or was located in close proximity to water courses, or abandoned meanders and oxbow lakes. The dominant vegetation types are mostly sedge- and moor-grass meadows, reed beds, and willow patches along streams.

(ii) Floodplain forest was located along the Elbe river, in sites with high groundwater level and regular flood cycles, forming a mosaic of wetter and drier patches. Prevailing trees are oak, poplar, elm and ash; typical plant communities are alder carrs and willow carrs, with treeless patches covered by reed and tall-sedge beds and wet meadows. Presence of seasonal and perennial pools or creeks by the banks of the Elbe River is typical of this habitat, which represents the wet side of the moisture gradient with closed canopy.

(iii) Mixed forest, the dry variant of the closed-canopy habitat, with oak- and oak-hornbeam woodlands; the other dominant species of these communities were lime, birch, spruce and pine.

(iv) Shrubby grassland was a savanna-like dry alternative of the open habitat. This habitat was dominated by grasses with scattered shrubs, mostly blackthorn and hawthorn (Fig. 3).

### Data collection

Each habitat type was represented by 3–4 spatially isolated sites, giving the total of 13 sites: wetland (4), floodplain forest (3), mixed forest (3), and shrubby grassland (3). The sites were located on average 3 km from one another. In each site we placed 4–10 camera traps, depending on the area of the site; each habitat was therefore monitored by 15–20 camera traps in total as follows: wetland (18), floodplain forest (19), mixed forest (15), and shrubby grassland (21). We used 73 UOVision type UV 535 Panda camera traps with infrared flash. The minimal distance between the traps within a site was 200 m and they were distributed so as to cover the range of conditions represented at a site, from the margins to the interior of the given habitat. The particular placement spots for the traps were chosen with consideration of the expected carnivores’ occurrence, i.e. mainly along animal trails, near water, along terrain depressions, etc. The traps were placed on trees approximately 0.5–1 m above the ground and we also had to consider possible human presence, so we chose places where we expected the least movement of people. The project started at the beginning of June 2015 and the results from the first complete year, until the end of May 2016, are reported here. The camera traps were in the field non-stop and data collection (photo downloading) was done at all sites approximately once a month. In total, we gathered over 900,000 photographs with the majority of them being empty or of non-target animal groups (such as ungulates, rodents, or occasionally birds).

### Data standardization

Because it was not possible to identify individual animals (especially on the night photographs, which were black and white because of the infrared flash), in order to infer data on abundances we standardized the data as follows. First, if the same animal was recorded as moving around on a series of subsequent photographs taken over less than two minutes, we considered this as one record. If such an individual was present for a long period of time without leaving the spot in front of the camera trap, for example resting, feeding or sleeping, we also considered that as one record. The data standardized in this way (termed ‘standardized records’), making up the total of 5011 records, were used for the analysis of patterns in daily activity.

For other analyses that addressed seasonal dynamics and habitat preferences, we used another standardization procedure. To reduce the possibility of bias caused by the repeated presence of an individual animal at the same camera trap, we only counted the presence of a particular species at each camera trap in a given day, disregarding the number of records (further termed as ‘standardized daily records’). After this standardization we were left with 3903 records of carnivores (i.e. 78.9% of the total number of 5011 records).

### Statistical analysis

Differences in the numbers of species among habitats were tested by using GLM models with Poisson distribution of errors. The effect of habitat and season on the number of standardized daily records of individual species was statistically tested only for those species with > 50 records (fox, marten, badger, least weasel), using a linear model with normal errors. Because the numbers of records at individual sites were divided by the numbers of camera traps taking pictures at the given time (accounting for the fact that some might not be functioning between two samples due to technical problems or theft until replaced), we could not use a GLM model with Poisson distribution of errors. The models were tested by step-wise removal of interactions or factors (Crawley 2007). The sites (*n* = 13) were treated as a random factor, and the factor ‘season’ was nested within the site to eliminate the effect of pseudoreplication.

The significance of interaction between the number of standardized records during day vs. night and season was tested by using GLM model with Poisson distribution of errors (Crawley 2007), and the significance of differences between cells according to Řehák and Řeháková (1986). There were two tests carried out for each species, one on standardized data as recorded, and the other one on the data related to the duration of day and night in particular seasons (using the ratio of absolute number of records in a season to the proportional length of the day in that season, averaged across the three months).

## Results

### Carnivore species richness

In total we recorded nine carnivore species in our study area: red fox (*Vulpes vulpes*), European badger (*Meles meles*), pine marten (*Martes martes*) and stone marten (*Martes foina*; these two species were merged into one group “marten”, due to the difficulty of recognizing them especially on the nocturnal black and white photographs), stoat (*Mustela erminea*), least weasel (*Mustela nivalis*), European polecat (*Mustela putorius*), European otter (*Lutra lutra*) and golden jackal (*Canis aureus*). The highest numbers of species occurred in wetland (7), followed by mixed forest and shrubby grassland (6), and the least in the floodplain forest (4), but these differences were not significant (GLM model with Poisson distribution of errors; df = 3, dev. = 2.09, P = 0.55). The most frequently recorded species were fox (n = 2069), marten (n = 1014) and European badger (n = 617), the species with the lowest number of records was otter (n = 7). Standardized numbers of species in respective habitats are shown in Table 1.

### Habitat preferences

Habitats with the highest standardized numbers of daily records pooled across all species were wetland (1279) and shrubby grassland (1014); fewer records were made in mixed (876) and floodplain forest (734) (Table 1).

**Table 1.** Standardized numbers of carnivore records in particular habitats in the four seasons. Data are summary numbers of records from all camera traps located in a given habitat, captured in a particular season.

	Spring	Summer	Autumn	Winter	Total
**Mixed forest**	**220**	**256**	**250**	**150**	**876**
fox	68	60	115	82	325
badger	92	89	86	31	298
marten	60	107	40	33	240
jackal			2	4	6
weasel			5		5
stoat			2		2
**Floodplain forest**	**173**	**123**	**234**	**204**	**734**
fox	39	53	140	130	362
marten	124	60	87	69	340
polecat	8	10	5	4	27
otter	2		2	1	5
**Wetland**	**230**	**269**	**372**	**408**	**1279**
fox	143	173	244	295	855
marten	76	72	106	107	361
stoat	1	9	12	2	24
weasel	3	3	6	2	14
badger	7	3	2		12
polecat		8	2	1	11
otter		1		1	2
**Shrubby grassland**	**214**	**286**	**287**	**227**	**1014**
fox	100	163	156	135	554
badger	85	96	76	50	307
marten	25	11	19	18	73
weasel	3	3	21	16	43
jackal	1	12	15	8	36
polecat		1			1
**Total (all habitats)**	**837**	**934**	**1143**	**989**	**3903**
fox	350	449	655	642	2096
marten	285	250	252	227	1014
badger	184	188	164	81	617
weasel	6	6	32	18	62
jackal	1	12	17	12	42
polecat	8	19	7	5	39
stoat	1	9	14	2	26
otter	2	1	2	2	7

Since we were interested in how the numbers of standardized daily records of the common carnivore species were affected by habitat and season, we first tested for the interaction of these two factors. This interaction was not significant for any of the species (fox: F = 0.41; badger: F = 0.39; marten: F = 1.05; least weasel: F = 1.64; df = 36, 45); therefore we tested for the effect of habitat and season separately. Habitat had a significant effect on the number of records of badger and marten, and marginally significant on fox (Table 2). Foxes were most often recorded in wetland (41% of all standardized daily records) and this difference was statistically significant (P < 0.05). Of all species, the red fox had the most even distribution among habitats. The records of marten were also evenly distributed, with the exception of shrubby grassland, where it was significantly less represented (Fig. 3). Badgers were only present in the dry habitats, shrubby grassland and mixed forest, with only 2% of records from wetland. Least weasel had the strongest, statistically significant preference for shrubby grassland with 69% of the records (both these species were missing from the floodplain forest). All the remaining species, for which the habitat preferences were not statistically tested due to a small number of records, revealed a strong preference for a certain habitat, stoat in floodplain forest (92%), jackal in shrubby grassland (86%) and otter (71%) and polecat (69%) in floodplain forest (Fig. 3).

**Table 2.** Effect of habitat and season on the numbers of standardized daily records of the four species with sufficient number of records. Tested by using linear regression with normal distribution of errors (df = 3, 48); n.s., not significant.

Species	Factor	F	P
fox	Habitat	2.15	< 0.1
	Season	2.92	< 0.05
badger	Habitat	12.07	< 0.001
	Season	0.53	n.s.
marten	Habitat	5.79	< 0.05
	Season	0.24	n.s.
least weasel	Habitat	5.79	< 0.05
	Season	2.60	< 0.1

### Seasonal dynamics

**Figure 4.**
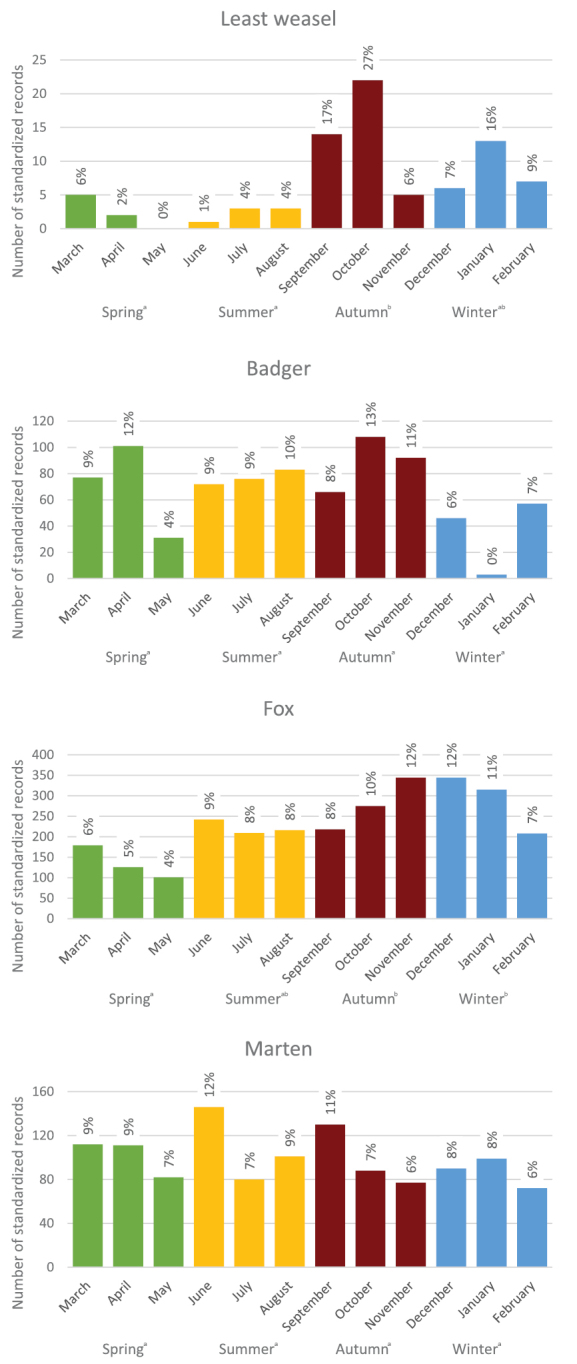
Seasonal dynamics shown for the carnivore species commonly occurring in the study area (with > 50 standardized daily records). The data were collected from June 2015 to May 2016, and the seasons are arranged in annual sequence for better illustration of seasonal dynamics. Seasons bearing the same letter are not significantly different from each other, based on linear model testing differences in the total number of records over the three months within the season. Values on top of the bars are percentages of the total number of records for a given species.

The total numbers of standardized daily records pooled across species and habitats were rather evenly distributed over seasons, reaching the highest values in the autumn (1143), lowest in spring (837), being very similar in summer and winter (934 and 989, respectively, Table 1). There were significant differences in the distribution of records of fox, marginally significant for least weasel, and these numbers did not significantly differ for badger and marten (Table 2). The number of red fox records increased throughout the year, from spring minima through stable numbers in summer and beginning of autumn, towards the highest numbers of records in November and December. The numbers of badger records fluctuate, with maxima in April and later on October and November, and markedly, but non-significantly, reduced activity over winter. Least weasel’s activity is greatest in autumn and very low in spring and summer. Marten was the only species with no obvious seasonal pattern (Fig. 4).

### Circadian activity

**Figure 5.**
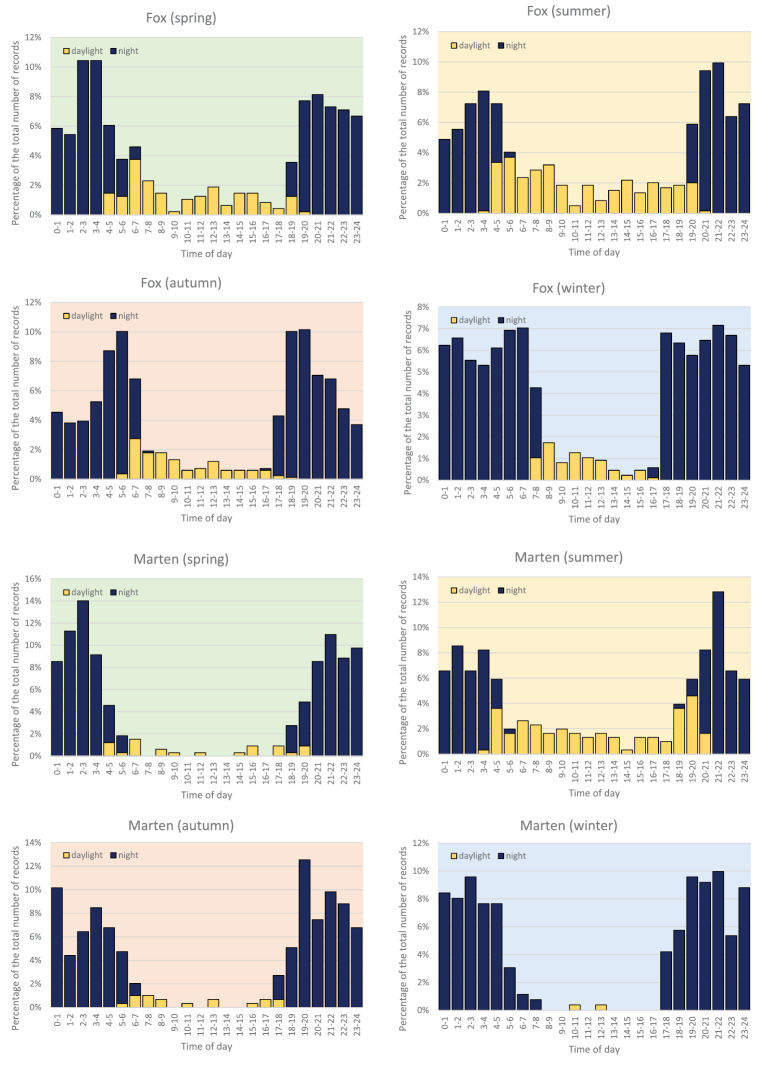
Circadian activity of fox and marten shown by season, expressed as the percentage of standardized records photographed at daylight and in the night. For badger, a whole-year summary is shown as the significant differences among seasons are due to it not occurring at daylight in winter.

Using exact time data recorded by the camera traps we analysed the circadian activity of all the species. Fox and marten were more often recorded during the day in the summer (with 33% and 34% of records, respectively), while in winter they were more active during the night (with only 8% and 1% of records recorded during the day). Badgers were only rarely photographed in the daylight (2%, 5% and 4% of records in spring, summer and autumn, respectively, and none in winter, Fig. 5). For these three most common species we tested, using the numbers of records standardized by the seasonal variation in day length, whether the percentage of records from day and night differed depending on the season. The differences observed for fox and marten were statistically significant, while those for badger were not (Table 3).

**Figure 6.**
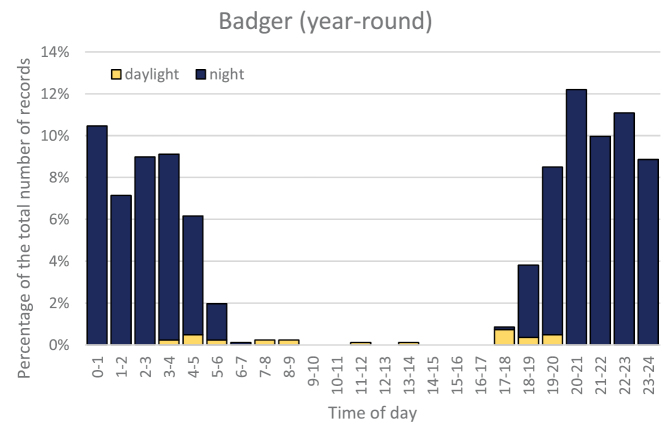
Continued.

**Table 3.** Interaction between season and the number of standardized records collated in daylight vs night, presented for the three most common carnivore species studied. The data were tested on contingency tables following Crawley (2007), with df = 3 for all tests; n.s., not significant. The analysis is based on the numbers of records standardized by the length of the day in particular seasons (using the ratio of absolute number of records in a season to the proportional length of the day in that season, averaged across the three months). Note that the least weasel is not included in the test because the number of records (n = 62) was too low for robust analysis.

Species	χ^2^	P
fox	19.08	< 0.001
marten	8.32	< 0.05
badger	1.89	n.s.

## Discussion

### Methodological assumptions

Most studies using camera traps focus on particular species, habitat type or topic. The majority of studies are on carnivores in forest habitats and most studies focus on population densities (McCallum 2013). While there are hundreds of studies based on camera traps published each year (over 200 papers in Web of Science categories ‘ecology’, ‘zoology’ and ‘conservation’ for 2016; WoS search as of 21 June 2017) papers quantitatively comparing the occurrence of carnivores in a range of habitats representing different levels of moisture and canopy openness are, to best of our knowledge, non-existent for temperate Europe. Our study started in June 2015 and covered all seasons with the traps permanently present throughout the year. We investigated species composition and structure in a range of habitats, representing the current central European landscape, the seasonal and circadian dynamics of all the species in all habitats and their habitat preferences.

Because it was not possible to identify individual animals on photographs, we did not attempt to estimate population densities and the quantitative comparisons were rigorously tested only interspecifically. We assumed that individuals of the same species behave in a similar way in different habitats or seasons, therefore the frequencies of captures do not systematically differ among these factors. Based on this assumption, we expect that a species with significantly more records in certain habitat or season is indeed more abundant in the respective habitat or season.

To estimate population densities, the capture-recapture technique is necessary (Karanth 1995, Karanth and Nichols 1998) if individual recognition is not possible (Wilson and Delahay 2001, Jennelle et al. 2002). However, in our study the capture-recapture method was not feasible for logistic reasons, including difficulties with obtaining permits for this kind of animal handling. While we were able to recognize some individuals repeatedly visiting locations of particular camera traps, we could not do so with certainty for different camera traps within- or among sites. With some other species such as badgers, individual recognition, which is crucial for abundance- and population-densities estimates (Trolle and Kery 2003), was not possible especially on black and white photos. Some authors argue that mathematical models can overcome the need for individual recognition (Rowcliffe et al. 2008, Shulman et al. 2016), but other authors invalidate these assumptions (Sollmann et al. 2013) and warn about a number of pitfalls (Foster and Harmsen 2012). Indeed, our data for one species suggest that population density estimates based on the number of records would really be biased – the 47 records of a golden jackal probably capture just one individual (Pyšková et al. 2016).

### Distribution and abundance of carnivore species in the Czech Republic

Out of 13 native carnivore species (notwithstanding the extinct European mink *Mustela lutreola* and four alien species) hitherto recorded in the Czech Republic (Anděra and Červený 2009, Pyšková 2017) eight were recorded in our study. Four of the native species that are absent from our record have rather restricted distribution in the country: gray wolf (*Canis lupus*), European lynx (*Lynx lynx*), wild cat (*Felis silvestris*) and brown bear (*Ursus arctos*); the latter species was recently repeatedly reported to cross the Polish or Slovakian borders (Anděra and Červený 2009). The remaining species, steppe polecat (*Mustela eversmanii*), is known from few records dispersed in the lowland regions of the country (Anděra and Gaisler 2012) and although it cannot be excluded at least in two areas of our study, until now it was not recorded. The majority of information on the distribution of carnivores comes from questionnaires and hunting statistics, compiled in approximately 10-year intervals (Anděra and Hanzal 1996, Anděra 2017a). The hunting data are not collected systematically, therefore their quality varies from one region to another, yet they provide a broad overall picture of how common individual carnivore species are in the Czech Republic.

The red fox and European badger are the most common carnivore species throughout the whole country and their occurrence is stable. The same holds for stoat and least weasel. The European polecat is also present in most grid cells, but its population densities have been declining slowly, and nowadays it is becoming rare or even extinct in some regions. Our results, with only 39 records of European polecat across all habitats throughout the year, seem to reflect this declining population trend reported by IUCN, which is also happening in neighbouring countries, Austria and Germany (Skumatov et al. 2016). Both species of marten are also common, forming stable populations. One species undergoing a significant change is the otter – it has become more common again in the past decades (Anděra and Hanzal 1996, Anděra and Červený 2009).

In our study, the most common species was the red fox. The data allow for quantifying the probability of its occurrence in a certain spot throughout the duration of the study. In total, there were 2096 standardized daily records of fox (i.e. 68% of all 3903 records pooled across species); taking into account that the maximum number of daily records from 62 camera traps over the whole year is 22,692 (the maximum number of daily records is reduced due to the possible malfunction of camera traps or theft between two sampling dates before replacement), there is a ~9% probability that the fox would be observed in a place where a camera was placed at least once a day. The red fox is not protected by law and can be hunted throughout the year without any restrictions in the Czech Republic (Regulation MZe ČR 245/2002 Sb). After eradication of rabies in the mid-2000s (Matouch et al. 2006, 2007), hunting represents the only means of regulating fox populations; however, this need not necessarily lead to lower population densities because foxes are reported to respond to hunting pressure by increasing their reproduction rate (Lozano et al. 2013).

Martens were the second most frequently captured carnivores. Since the hunting statistics utilize the same species merging (pine and stone marten) methodology as we did, our results are directly comparable and support the belief that these species are common in the Czech Republic. The same holds for badger, another frequently recorded species in our area; it is considered very common, widespread and with the population tending to increase in the country (Anděra and Červený 2009).

As for other species, the weasel and stoat are also considered to be common, but data on animals that had been shot are missing since neither species is on the hunted species list. The fairly low numbers of records of these species (Table 1) can be due to several factors. First, weasels and stoats travel shorter distances, their home ranges are smaller and show more restricted habitat preferences (stoats prefer wetter habitats, weasels open ones; Fig. 3; Hunter 2011, McDonald et al. 2016), which eliminates some of our trapping locations. Second, a small body size can cause the animal to be less visible, allowing it to pass by uncaptured by the camera trap, including walking through bushes rather than on a path which is preferred by larger animals. It is therefore likely that the recording bias was higher for these species and the captures may reflect less reliably their true population trends. This is, however, not caused by the inability of the traps to capture them due to their small body size because the traps were activated by even smaller animals such as mice or even larger insects.

### Alien species

The only non-native species recorded in the study area is the golden jackal. The status of this species is unclear, as it cannot be considered invasive, but we also do not consider it native because its historical range in Europe never reached these latitudes at least during the Holocene (Pyšková et al. 2016). Of the invasive species of carnivores in the Czech Republic – raccoon (*Procyon lotor*), racoon dog (*Nyctereutes procyonoides*) and American mink (*Neovison vison*) – none were recorded during the year. This was not surprising for the raccoon, which is not widespread in the country as yet, but it was surprising for the American mink, which started spreading rapidly in the 1990s and is now reported as widespread throughout all regions of the Czech Republic. Similarly, raccoon dogs, also reported as widespread by national grid mapping (Anděra and Červený 2009, Anděra 2017b), are believed to be opportunistic generalists colonizing almost any location where water, food and resting opportunities are available. The absence of raccoon dog in our study area is unlikely to be caused by interspecific competition with foxes and badgers as these species were shown to permanently coexist in many regions of their syntopic occurrence (Sidorovich et al. 2000, Drygala and Zoller 2013). One feasible explanation could be the shyness of the species. While in Japan they wander close to human settlements, in Europe they tend to avoid them (Kauhala 1995, Drygala et al. 2008, Kauhala and Salonen 2012). Moreover, raccoon dog inhabits middle elevations in the Czech Republic and is quite rare below 200 m a.s.l, i.e. the altitude of our study area (Anděra and Gaisler 2012). The obvious contradiction between the widespread occurrence in grid cells at the national scale and absence from our local records raises the question to what extent can we extract the information on carnivores from the traditional sources (hunting statistics and questionnaires).

### Habitat preferences, seasonal dynamics and circadian activity of the carnivore species studied

The red fox preferred wetlands in our study, but it is a habitat generalist, with other habitats represented almost equally. This agrees with the fact that fox was the most common carnivore in our study. It is not restricted by particular habitat preferences, therefore it can prosper anywhere, even in cities, where their population densities sometimes reach higher numbers than in natural habitats (Bateman and Fleming 2012). The opportunistic omnivore diet of this species allows for a low level of habitat specialization, especially in the Czech cultural landscape with plentiful food sources (for example numerous records of hares in our camera traps). Martens were represented equally in all habitats except shrubby grassland, reflecting the pine marten’s preference for more wooded areas (Anděra and Horáček 2005). Based on habitat preferences of the marten species occurring in the Czech Republic, we assume that most of the animals were individuals of the pine marten. Badgers were mainly present in the dry habitats and avoided wetlands and floodplain forests where they cannot dig their deep burrows in wet soils. Only a few records of badgers were made in wetlands, but we assume the individual animals were just passing through, not being permanent residents, as they were not photographed repeatedly. The weasel was mostly present in the shrubby grassland, reflecting its preference for open habitats (McDonald et al. 2016). As for the other species whose habitat affiliations were not tested statistically due to the low number of records, all of them occurred in supposed habitats typical for them, confirming they prefer certain environmental conditions.

The quantitatively documented patterns in the seasonal circadian activity were most pronounced in the red fox and can be related to reproductive period. Foxes were recorded with a significantly lower frequency in the spring, which is the season of cub rearing, when the mother spends more time in the den with the young, and the father staying nearby to help with care of the cubs (Macdonald 1979). During the rest of the year the activity of foxes was quite evenly distributed and increased again in November and December – this could be related to a greater effort needed to secure food. Quite the opposite results were shown for the badger, which were not active at all during heavy snowfall and frost. This is because in harsh conditions badgers retire to winter sleep (Matyáštík et al. 2000). Martens do not show any significant variation in seasonal dynamics, since they are habitat and food generalists and thus are active throughout the year; lower numbers in May can be also related to the rearing of the young.

Most of our carnivores have crepuscular or nocturnal activity (Anděra and Červený 2009). Foxes were most active early in the morning (4–5) and evening (19–22) hours, but the pattern varied with respect to season. In summer, the foxes are more active during the daylight – this is caused by the fact that when the female is rearing the cubs, she stays out longer to hunt (Harris and Yalden 2008). It also needs to be noted that for all species except the red fox, the differences in proportions of records taken at daylight vs. night disappear, or are smaller, if the different length of the day in particular seasons is taken into account. The fox is not strictly nocturnal in any season, unlike the badger, which was captured in daylight in only 2% of all the records. Martens were more nocturnal than foxes, although in the summer they were also more active during the day (about one third of the records), while in winter they were almost exclusively active at night. The weasel and stoat were both more active during the day, confirming common knowledge (Anděra and Horáček 2005, Samson and Raymond 2009), the polecat was recorded mostly at night, although not entirely, and the golden jackal was most active early in the morning (Pyšková et al. 2016).

## Conclusions

Our study is the first that provides systematically collected quantitative data, made possible by employing a relatively new technology, to assess the frequency of occurrence of central-European carnivore species. In general, our results confirm the known historical, largely anecdotal information on the ecology of the species as reported in the faunal literature. Although there are no quantitative historical data to which our results can be compared, it appears that ecological preferences of the carnivores in our study system have not changed much, if at all, while the central-European landscape has changed immensely in the past century due to human activities. The landscape has turned into a mosaic of human settlements, infrastructure, industrial or agricultural land and patches of semi-natural habitats, with even the latter not free from the influence of people in the form of management or tourism.

The results presented in our study further indicate that carnivores are fairly frequent in such a modern landscape, and the majority of species successfully adapted to the changes that have occurred over the last century. Since the industrial revolution, agricultural production, as well as urbanization and other human-related disturbances, have significantly increased. However, in last few decades these trends were complemented by decreasing direct human pressure (including hunting) driven by the decline of the traditional rural way of life and increasing areas of forests and shrubs due to decreasing needs for food production in less fertile regions. It is possible that due to these changes, the landscape was becoming increasingly more suitable for wildlife. More studies are needed for confirmation of the broader generalizations of these trends, but that the mesocarnivores are successfully inhabiting the open landscape is good news, even considering the limitations of our regional-scale study.
